# Toothbrush Abrasion of Restorations Fabricated with Flowable Resin Composites with Different Viscosities In Vitro

**DOI:** 10.3390/ma14216436

**Published:** 2021-10-27

**Authors:** Yuko Miyano, Masaya Suzuki, Koichi Shinkai

**Affiliations:** 1 Advanced Operative Dentistry-Endodontics, Graduate School of Life Dentistry at Niigata, The Nippon Dental University, Niigata 951-8580, Japan; miyanou@ngt.ndu.ac.jp; 2 Department of Operative Dentistry, School of Life Dentistry at Niigata, The Nippon Dental University, Niigata 951-8580, Japan; collagen@ngt.ndu.ac.jp

**Keywords:** flowable resin composite, rheology, three-body wear, toothbrush wear, viscosity

## Abstract

The purpose of this study was to examine toothbrush-induced abrasion of resin composite restorations fabricated with flowable resin composites of different viscosities in vitro. In this study, six types of flowable resin composites with different flowability (Beautifil Flow F02, F02; Beautifil Flow F10, F10; Beautifil Flow Plus F00, P00; Beautifil Flow Plus F03, P03; Beautifil Flow Plus X F00, X00; and Beautifil Flow Plus X F03, X03) were used. For the toothbrush abrasion test, the standard cavity (4 mm in diameter and 2 mm in depth) formed on the ceramic block was filled with each flowable resin composite (n = 10) and brushed for up to 40,000 strokes in a suspension containing commercial toothpaste under the conditions of 500 g load, 60 strokes/min, and 30 mm stroke distance. After every 10,000 strokes, the brushed surface of the specimen was impressed with a silicone rubber material. The amount of toothbrush-induced abrasion observed on each impression of the specimen was measured using a wide-area 3D measurement device (n = 10). The viscosity was determined using a cone-and-plate rotational measurement system. Because of the effect of different shear rates on viscosity and clinical use, the values 1.0 and 2.0 s^−1^ were adopted as data (n = 6). In this study, the results of the toothbrush abrasion test demonstrated no significant differences in the amount of toothbrush-induced abrasion among flowable resin composites used (*p* > 0.05). No significant correlation was reported between toothbrush-induced abrasion and viscosities of flowable resin composites.

## 1. Introduction

The mechanical properties of resin composites are influenced by sizes, shapes, and contents of filler particles as well as the type of resin matrix. The wear resistances of resin composites are affected by the filler particle distribution and degree of polymerization of the resin matrix [1,2]. Currently, the commercially available universal resin composites contain fillers at a rate of ~60–80 wt% [3]. Resin composites composed of hybrid fillers, which contain multiple fillers of various sizes, have demonstrated high wear resistance at the occlusal surface, which is subjected to severe stresses during chewing. Multiple in vitro and in vivo studies reported that the addition of variable-sized fillers [4,5,6,7,8,9] and nanosized filler particles [10,11] improved the wear resistance of universal resin composites, which has not raised concerns as a serious problem in clinical practice [12,13].

Flowable resin composites are frequently used in clinical practice because they are convenient for filling tooth cavities using a direct application syringe. When a flowable resin composite was first used in the clinical setting, its application was limited to the lining and base of the cavity because its mechanical properties were inferior to those of the universal resin composite [14]. However, the mechanical properties of flowable resin composites have been improved by adopting new monomers and nanosized filler particles, and they are applied to various cavities in clinical settings. [12,13,15,16,17]. Because of mechanical improvements, certain products of flowable resin composites demonstrate high wear resistance equivalent to that of universal resin composites and are used to restore occlusal cavities. Shinkai et al. [11] reported that the wear resistance of flowable resin composites containing nanosized or spherical fillers was equivalent to that of universal resin composites based on the results of three- and two-body wear testing.

Commercially available flowable resin composites are classified into high-, medium-, and low-flow types as per their viscosity or flowability [14,18,19,20,21]. The application of each flow type to various cavities is subjected to the form, size, and position of the cavity and the purpose of application. The viscosity of resin composite depends on the components of the resin matrix, in addition to the size, shape, content, and surface treatment of filler particles [22,23]. Previously, several studies confirmed the effects of these factors on the mechanical properties of resin composites [24,25,26]. However, the effect of the viscosity of flowable resin composite on the toothbrush-induced abrasion of flowable resin composite restorations has not been clarified to date.

In previous studies on the rheological properties of flowable resin composites, the dynamic oscillatory shear test has been used frequently owing to the viscoelasticity of flowable resin composites [27,28,29,30]; however, the apparent viscosity, which is determined from the ratio of shear stress to shear rate, is considered the most straightforward index of the viscosity of flowable resin composites because they are non-Newtonian fluids whose flow characteristics change under different shear test conditions. Therefore, in this study, we confirmed the apparent viscosities of flowable resin composites using a cone-and-plate rotational viscometer.

This study aims to examine the toothbrush abrasion of resin composite restorations fabricated using the different viscosity flowable resin composite in vitro and the correlation between the viscosity and the amount of toothbrush abrasion of flowable resin composites. The null hypothesis of this study was that a difference in viscosity of the flowable resin composites would not influence the toothbrush abrasion of resin composite restorations.

## 2. Materials and Methods

### 2.1. Materials Used

Table 1 lists the flowable resin composites used in this study.

Six flowable resin composites (Beautifil Flow F02, F02; Beautifil Flow F10, F10; Beautifil Flow Plus F00, P00; Beautifil Flow Plus F03, P03; Beautifil Flow Plus X F00, X00; and Beautifil Flow Plus X F03, X03) manufactured by the same company (Shofu Inc., Kyoto, Japan) were used. For the surface treatment of the ceramic cavity, a bonding agent (Beautibond Universal; Shofu Inc., Kyoto, Japan) and ceramic primer (Beautibond Universal Porcelain Activator; Shofu Inc., Kyoto, Japan) were used. Ceramic blocks (Vitablocs Mark II; Hakusui Trading Co. Ltd., Osaka, Japan) were used to prepare specimens for the wear test because the hardness of ceramic blocks is higher than that of the resin composites and enamel.

### 2.2. Preparation of the Wear Test Specimens

The truncated cone shape cavity (4 mm in diameter and 2 mm in depth) was prepared on the central flat surface of the ceramic block (Vitablocs Mark II) with a barrel-shaped diamond point (No. 144; Shofu Inc., Kyoto, Japan). The cavities were cleaned using 40% phosphoric acid gel (K-Etchant; Kuraray Noritake Dental Inc., Tokyo, Japan) for 10 s, followed by complete rinsing and drying. The bonding agent was applied to the cavities for 10 s, and then the ceramic primer was rubbed on the cavity surfaces for 5 s. After the adhesives were applied, the cavities were first air-dried under low pressure for 3 s and then under high pressure according to the manufacturer’s instructions and finally photopolymerized for 10 s using a light-curing unit (Candelux, Morita Corporation, Kyoto, Japan). For each filling, each flowable resin composite was filled in the cavities incrementally twice and photopolymerized for 20 s using the light-curing unit. After storing the specimens in a thermo-hydrostatic apparatus (37 °C, 95% humidity) for 48 h, the resin surface of the specimens was polished to flatten them using a 1500-grit SiC paper. This delayed finishing and polishing of the filled resin composite to flatten the surface of the specimen after completing expansion through water absorption prevents extra expansion, which may enable one to accurately measure the amount of abrasion. Ten specimens were prepared for each flowable resin composite. This procedure of specimen preparation was based on our previous studies [31,32].

### 2.3. Toothbrush Abrasion Test

The toothbrush abrasion test was conducted using an abrasion tester (K236; Tokyo Giken, Inc., Tokyo, Japan) under the following conditions: 500 g load, 60 strokes/min, and 30 mm stroke width. After the specimen was fixed to the fixing table and the toothbrush was set as the moving arm on the abrasion tester, the toothbrush was placed on the restoration in the specimen. During the abrasion test, the toothbrush slid with reciprocating motion under the abovementioned conditions. In this study, the toothbrushes and medium used were Prospec Toothbrush Adult Hard (GC Corporation, Tokyo, Japan) and White & White (Lion Corporation, Tokyo, Japan), respectively, and they were replaced with new materials every 10,000 strokes. The impression of the brushed resin surface on the specimen was taken using an additive silicone rubber impression material (Exafine Injection; GC Corporation, Tokyo, Japan) after every 10,000 strokes, and the specimens were subjected to a toothbrush abrasion test until 40,000 strokes. The wear volume was calculated by measuring the 3D shape of the abraded resin surface on each impression using a wide-area 3D measurement device (VR-5000; Keyence Corporation, Chicago, IL, USA.).

### 2.4. Measurement of Viscosity

At room temperature (25 °C), the viscosity (mPa·s) of each flowable resin composite was measured using a cone-and-plate rotational viscometer (RVDV2TCP; Eikoh Seiki, Tokyo, Japan). Each flowable resin composite paste of 1 cc was placed on the measuring plate of the viscometer and left for 10 min until the flowability was regulated; subsequently, the viscometer rotated at a shear rate of 0.1–2.0 s^−1^ to obtain the flow curves of shear stress (Pa) and viscosity (mPa·s). Viscosity was determined six times for each flowable resin.

### 2.5. Statistical Analysis

The data from the viscosity test were statistically analyzed using one-way analysis of variance, and a post hoc test was performed using Tukey’s test to confirm significant differences among the viscosities of six flowable resin composites because the data showed homoscedasticity. The data from the toothbrush abrasion test were statistically analyzed using the Kruskal-Wallis and Steel-Dwass tests to confirm significant differences among the wear volumes of six flowable resin composites after 10,000, 20,000, 30,000, and 40,000 strokes and among the wear volumes after 10,000, 20,000, 30,000, and 40,000 strokes per material because the data did not show homoscedasticity. Using linear regression analysis, the correlation between viscosities and wear volumes of flowable resin composites was determined.

### 2.6. Scanning Electron Microscopy Observation

Observing the microstructure of the surface of the specimens after conducting the wear test is necessary to examine the size, shape, and distribution of the fillers on the respective flowable resin composite and analyze the effects of these characteristics on the wear resistance of flowable resin composite restorations. Therefore, to determine the microstructure morphology of the abraded resin composite surface of each material, the abraded surface of each flowable resin composite after 40,000 strokes was observed using scanning electron microscopy (SEM).

## 3. Results

### 3.1. Measurement of Wear Volume Using a Toothbrush Abrasion Test

**Figure 1:**
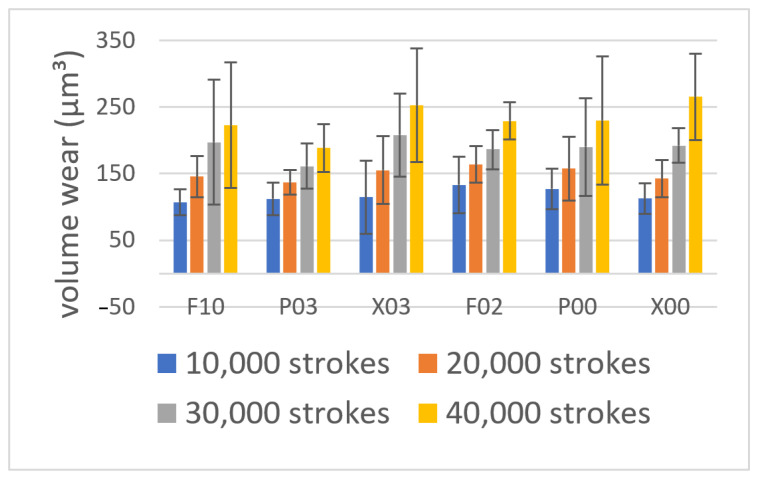
The wear volume of each flowable resin after the toothbrush abrasion test. The wear volume shows a tendency to increase linearly with the number of strokes during the test.

**Figure 2:**
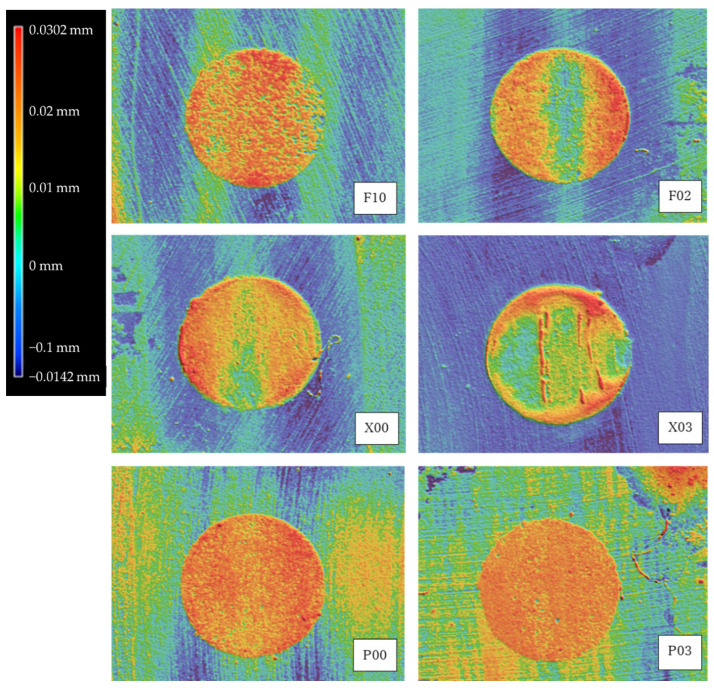
Representative color images showing the wear depth in each sample after 40,000 strokes. P00, P03, and F10 show complete wear on the surface of the resin composite, whereas X00, X03, and F02 show partial wear at the peripheral parts of the surface of the resin composite.

**Figure 3:**
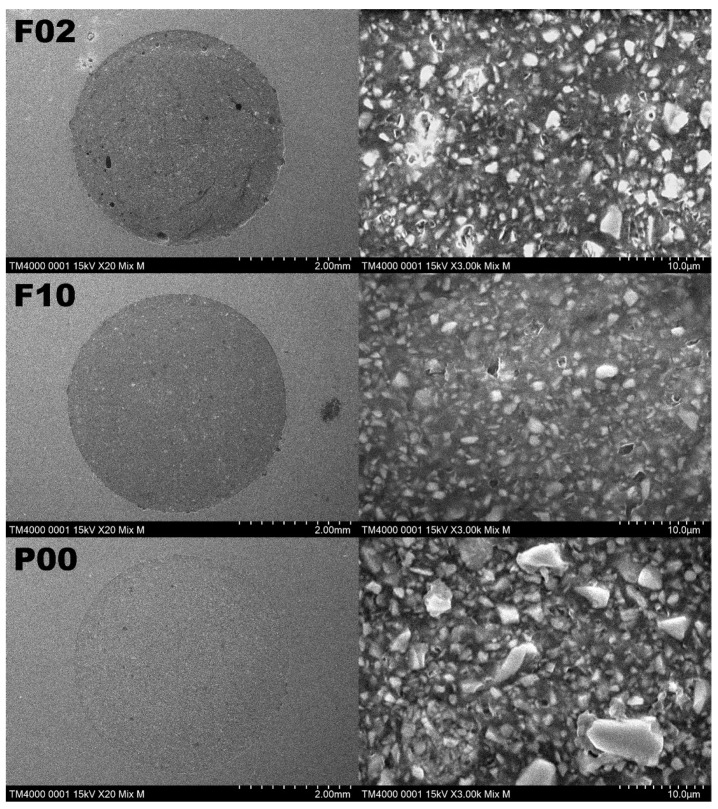

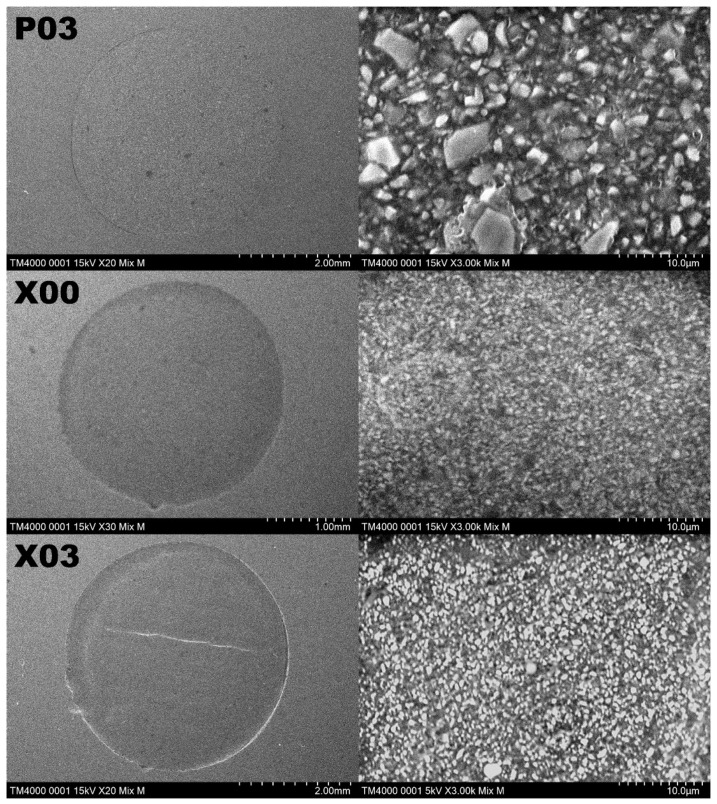
Scanning electron microscopic photographs of the composite materials after the wear test (magnifications: 20× (left) and 3000× (right)). The filler sizes of P03 and P00 were the largest, followed by those of F10, F03, X03, and X00, in that order. Certain indentations and convexities can be observed on the surface of the abraded specimens of P00, P03, F02, and F10, which may have resulted from filler dropout. However, all filler particles of X00 and X03 are extremely small, and the abraded surfaces are relatively flat.

Figure 1 shows the wear volumes of flowable resins after the toothbrush abrasion test. The wear volume of each flowable resin tended to linearly increase with the increase in the number of strokes during toothbrush abrasion. However, after the corresponding strokes, no significant differences were detected among flowable resins (*p* > 0.05). Nevertheless, all flowable resins demonstrated a significant difference between the 10,000- and 30,000-stroke abrasions (*p* < 0.05) and between the 10,000- and 40,000-stroke abrasions (*p* < 0.01). The flowable resins except P00 demonstrated a significant difference between the 20,000- and 40,000-stroke abrasions (*p* < 0.05). Figure 2 shows the representative color images of wear depth in the case of each sample after 40,000 strokes. P00, P03, and F10 demonstrated complete wear on the surface of the resin composite, whereas X00, X03, and F02 demonstrated partial wear at the peripheral parts of the surface of the resin composite. Figure 3 shows the SEM images of each material after the wear test. The filler sizes of P03 and P00 were relatively large, followed by those of F10, F03, X03, and X00 in this order. Certain indentations and convexities were observed on the surface of abraded specimens of P00, P03, F02, and F10, which may be attributed to filler dropout. However, all the filler particles of X00 and X03 were considerably small, whereas the abraded surfaces were relatively flat.

### 3.2. Measurement of the Viscosity of Flowable Resins

**Figure 4:**
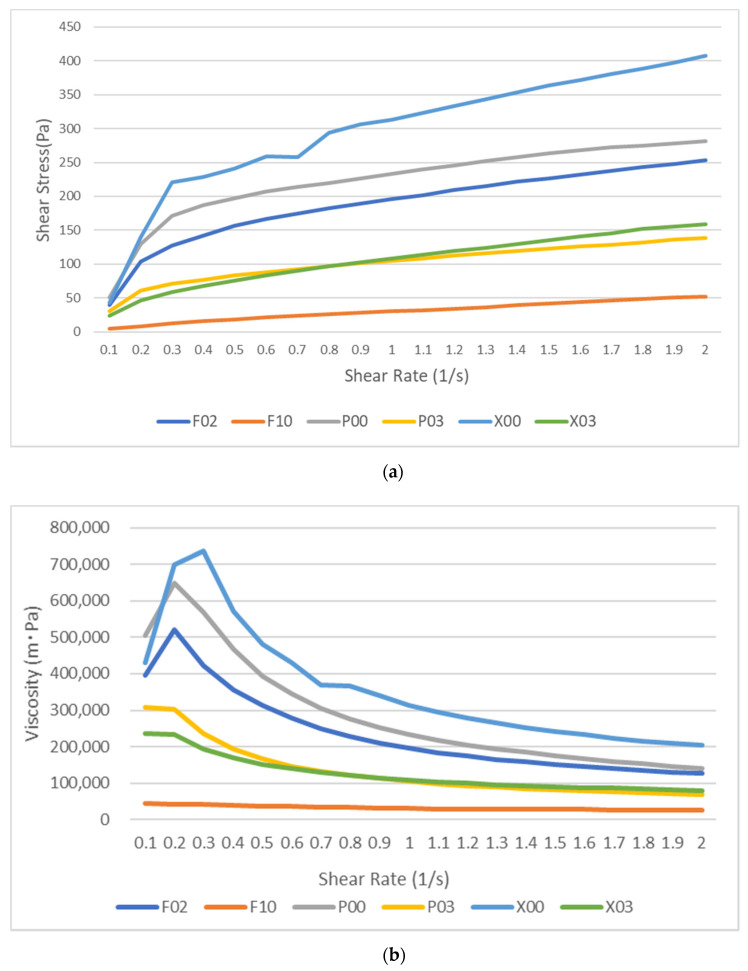
The average values of the shear stress (**a**) and viscosity (**b**) for each sample of flowable resin composite. The shear stress and viscosity values of all the flowable resins quickly increased on a curve until the shear rate of 1.0 s^−1^, whereas their increase became almost constant with a linear gradient after the shear rate of 1.0 s^−1^.

**Figure 5:**
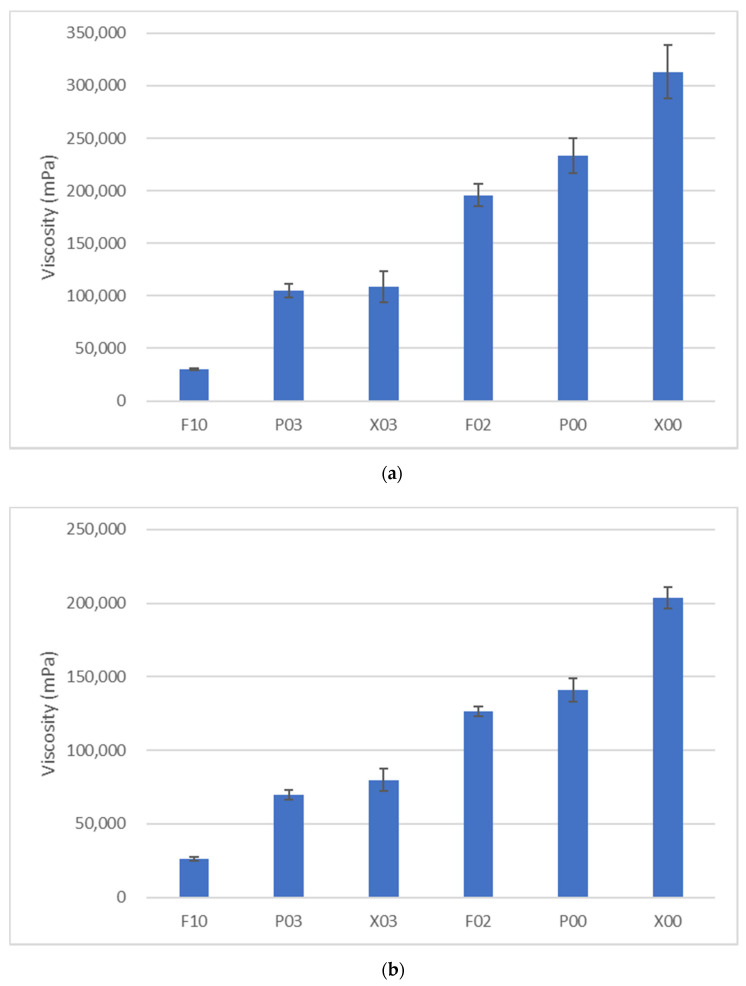
The viscosity values of the flowable resins at shear rates of 1.0 s^−1^ (**a**) and 2.0 s^−1^ (**b**). Regardless of shear rate, the viscosity value of the flowable resins was highest for X00, followed by P00, F02, X03, P03, and F10, in that order.

Figure 4 shows the viscosities of each flowable resin composite at different shear rates. The shear stress and viscosity of all flowable resin composites increased quickly with a curve until the shear rate reached 1.0 s^−l^, whereas their increase became almost a constant linear gradient after the shear rate of 1.0 s^−1^. In this study, we focused on the flow behavior of the flowable resin immediately after the application of shear stress. Because of the effect of shear rates on viscosity, we used data at the shear rates of 1.0 and 2.0 s^−1^. Figure 5 shows the viscosity values of flowable resins at the shear rates of 1.0 and 2.0 s^−1^. Regardless of shear rate, the viscosity values of flowable resins were higher in the following order: X00 > P00 > F02 > X03 > P03 > F10. Among all shear rates, significant differences in viscosity were observed for all the flowable resin composites except F10 (*p* < 0.01). Significant differences in viscosity were detected among all flowable resin composites except between P03 and X03 at a shear rate of 1.0 s^−1^ (*p* < 0.01). These differences were detected between F02 and P00 and between P03 and X03 at a shear rate of 2.0 s^−1^ (*p* < 0.01).

### 3.3. Linear Regression Analysis of Viscosity and Wear Volume after the Toothbrush Abrasion Test of 40,000 Strokes

**Figure 6:**
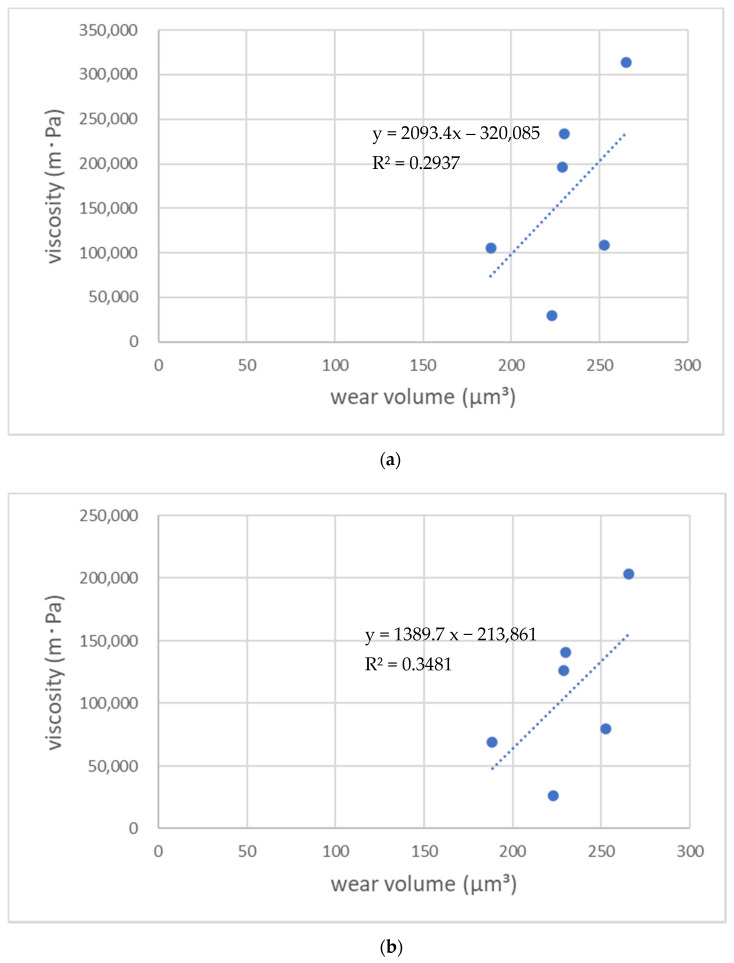
Correlation between the viscosity and wear volume values of the flowable resin composites at shear rates of 1.0 s^−1^ (**a**) and 2.0 s^−1^ (**b**). A weak positive correlation was observed at the shear rate of 2.0 s^−1^ (R^2^ = 0.3481), whereas no correlation was observed at the shear rate of 1.0 s^−1^ (R^2^ = 0.1815).

Figure 6 shows a correlation between viscosity and wear volume after the toothbrush abrasion test for each flowable resin. The results of the linear regression analysis demonstrated no significant correlation between the amount of tooth brushing wear and viscosities at each shear rate of the flowable resin composites used in this study (*p* > 0.05); however, a weak positive correlation was recognized between the viscosity and wear of the flowable resins at shear rates of 2.0 s^−1^ (R^2^ = 0.3481) and 1.0 s^−1^ (R^2^ = 0.2938).

## 4. Discussion

### 4.1. Viscosity of the Flowable Resin Composites Tested

Previously, studies reported that factors that influence the viscosity of resin composites were the resin matrix components (ratio and type of each component); the size, shape, and content of the filler; and the surface treatment of the filler [22,23,31]. The pairs F02 and F10, P00 and P03, and X00 and X03 are each composed of the same monomers. The filler contents and viscosities of F02, P00, and X00 are higher than those of F10, P03, and X03, respectively. Hence, in this study, the viscosities of flowable resin composites used were related to the filler contents, and not the base monomers. Previously, studies [22,23] reported that the viscosity of flowable resin composites increased with an increase in filler content, which agrees with the current results. In general, the addition of more particles to a Newtonian fluid causes interactions among particles. This interaction is decomposed by shear stress, and the flow behavior shows the shear thinning characteristics. In terms of this rheological property, Lee et al. [23] reported that viscosity increases as a function of shear rate when the contents of fillers exceed 30 wt%. Furthermore, this interaction occurs among filler particles and between filler particles and matrices in polymeric materials [32]. Owing to this interaction, a maximum filler content exists for each combination of matrix and filler particle [23]. Therefore, the viscosity of the flowable resin composite appears to be affected by both the contents of filler particles and the interaction between the filler particles and the resin matrix. Previously, studies reported that, when they contained the same amount of filler particles, the viscosity of resin composites containing small filler particles was higher [22,23]. After the wear test, the SEM images of X00 and X03 in Figure 3 demonstrate that both resin composites contain spherical fillers, and the filler particles in X00 are smaller than those in X03. These results indicate that the more viscous resin composites contained smaller fillers, which agrees with the results of previous studies. However, by comparing the viscosities of F02, F10, P00, and P03, which contained irregularly shaped fillers, we reported that the viscosities of F10 and P03, which contained smaller fillers, were lower than those of F02 and P03, unlike the result for the flowable resins containing spherical fillers. Viscosity decreases when the particle size distribution increases, and the same tendency was reported in products having irregularly shaped fillers. In this study, the results obtained by measuring the viscosities of flowable resins suggest that the viscosities of flowable resins may be influenced by filler characteristics such as content, particle size, and particle size distribution.

In this study, bisphenol A-glycidyl methacrylate (Bis-GMA) and triethylene glycol dimethacrylate (TEGDMA) were present in all flowable resins. Bis-GMA exhibits high mechanical strength after polymerization; however, it shows a remarkably high viscosity of ~1200 Pa-s [33]. Owing to the reduction in viscosity, TEGDMA, which has a viscosity of ~0.01 Pa-s, is commonly added as a diluent monomer. Because the viscosity of the resin matrix affects the filler content [34], controlling the viscosity could be important to provide sufficient physical properties to the resin composite. The viscosity of 2,2′-bis-(4-methacryloxy polyethoxyphenyl) propane (Bis-MPEPP), which is partially composed of X00 and X03, is approximately one-tenth of that of Bis-GMA [35]. The viscosity test results demonstrated that the viscosities of X00 and X03 tended to be higher than those of other fillers. TEGDMA is known to cause disadvantages, such as high water absorption, low physical properties, and low color stability, in resin composites. The higher the amount of TEGDMA used as a diluent, the more adverse effects it has on the resin matrix. Kalachandra et al. [36] reported that the use of a low-viscosity bifunctional monomer as an alternative to TEGDMA reduced the negative effects on the resin matrix. We consider that, owing to the addition of Bis-MPEPP, the additional amount of TEGDMA in X00 and X03 may be less than that in the other flowable resins. Consequently, the viscosities of X00 and X03 might have become slightly higher than those of other flowable resins.

### 4.2. Toothbrush Abrasion of the Flowable Resin Composites Tested

In the toothbrush abrasion tests in this study, a toothpaste containing calcium bicarbonate was used as the medium. Owing to their low hardness, calcium carbonate and baking soda are assumed to be safe as brushing media for enamel and dentin [37,38]. However, the calcium bicarbonate particles between the toothbrush and surface of the polymerized resin composite abrade the polymer matrix, causing the filler particles to fall off. Both toothbrush abrasion and viscosities of resin composites are affected by composition. In addition to the abovementioned factors, the distribution of fillers and degree of polymerization of resin matrix are important factors for controlling wear resistance. The results of this study indicate no correlation between filler content and toothbrush abrasion. Many studies that examined the correlation between filler content and wear resistance of resin composites [39,40,41,42] reported that increasing the filler content may improve wear resistance. However, in recent years, the composition of resin composites has been diversified; multiple studies demonstrated differences in the wear properties of various restorative materials [25,26,43,44], suggesting the influences of factors other than filler content on the wear resistance of resin composites.

The filler sizes of flowable resin composites, as shown in Table 1, are the average values announced by the manufacturer. However, in the SEM images, a considerable variation of filler size in each material was observed. Based on the SEM images shown in Figure 3, the maximum diameters of fillers and clusters were as follows: P00 ≥ P03 > F02 ≥ F10 > X03 ≥ X00. The results of the abrasion test demonstrated that X00 and X03 exhibited greater wear volume than other flowable resin composites. The maximum filler diameter of X00 and X03 is ~1 µm, which is smaller than those of other samples. The filler particles in both X00 and X03 are spherical and of the minifill type (particle size, 0.1–1 µm), which has less variation in size. Previously, studies reported that resin composites composed of nanofillers of <0.01 µm in size demonstrated superior wear resistance, whereas those composed of submicron fillers ranging in size from 0.4 to 1 µm tended to demonstrate various wear resistances [24,25]. Therefore, the wear resistance of resin composite composed of fillers of various sizes of >0.1 µm is influenced by other factors such as filler shape and resin matrix composition.

The wear volumes of P00, P03, F02, and F10, which contained irregularly shaped fillers, were less than those of X00 and X03, which contained spherical fillers. Compared with spherical fillers, the irregularly shaped fillers may strongly adhere to the resin matrix owing to the mechanical interlocking force. Hence, adhesion between the irregularly shaped fillers and resin matrix may be increasingly resistant against strains induced by long-term wear stress. Moreover, the irregularly shaped fillers may lead to strong chemical adhesion to the resin matrix owing to the large adhesive interface as compared with that of spherical fillers. Vogel et al. [25] examined the effect of filler shapes with equal particle sizes on the wear resistance of resin composites and reported that the resin composite containing irregularly shaped fillers demonstrated higher wear resistance than that of resin composites containing spherical fillers, which agrees with the results of this study. Moreover, they assumed that the filler component affected the wear resistance of the resin composite, whereas this study suggested that the filler component has no effect on the wear resistance of the resin composite because the flowable resin composites used contained the same component fillers, which are surface reaction-type prereacted glass ionomers and glass.

After the toothbrush abrasion test of 40,000 strokes, the wear volumes of X00 and X03 were larger than those of other resins, and localized wear at the cavity margin was observed in SEM images (Figure 3). As Bis-MPEPP, which is included in the compositions of X00 and X03, exhibits relatively low Knoop hardness [45], the localized wear observed in X00 and X03 specimens might have caused the convergence of repeated abrasion stresses at the margin of the cavity. The amount of Bis-MPEPP in X03 is assumed to be higher than that in X00 because the wear of X03 was more pronounced at the cavity margin. This indicates a relationship between the type of resin matrix monomer and wear resistance of the flowable resin composite. As mentioned previously, TEGDMA has the disadvantage of inducing a decrease in the mechanical properties of the resin matrix because of high water absorption [16,17,31,32,46]. However, Kawai et al. [44] confirmed that increasing the amount of TEGDMA resulted in a reduction in the amount of wear by performing a toothbrush abrasion test on the resin composite containing Bis-GMA and TEGDMA. From this study, we speculate that increasing the wear resistance of the resin composite, followed by increasing the amount of TEGDMA, may cause the property of TEGDUMA to improve the degree of polymerization and amount of fillers. In accelerated tests such as the toothbrush abrasion test used in this study, the water absorption by the resin matrix in a short time period might be low and might not have affected the wear resistance of flowable resin. Hence, in this study, TEGDMA might contribute to the improvement of the wear resistance values of flowable resin composites.

### 4.3. Correlation between Viscosity and Toothbrush Abrasion

The results of statistical analysis demonstrated no significant correlation between the viscosity and wear volume of flowable resins. Therefore, our null hypothesis that no correlation exists between the viscosity and wear resistance values of flowable resins in toothbrush abrasion was confirmed. However, this study has potential limitations. For commercializing flowable resins, the properties are determined by the manufacturer. Therefore, it is difficult to directly determine the effect of small differences in the composition of composites on viscosity and wear resistance. As mentioned previously, because multiple factors influence the viscosity and wear resistance values of flowable resins, many confusing factors exist. Therefore, in this study, these factors might in a complex manner influence the viscosity and wear resistance values of flowable resin composites. Consequently, this study might not demonstrate any significant correlation between viscosity and wear resistance. From the results of this study, we confirmed that the wear resistance values of flowable resin composites from the toothbrush abrasion test were not affected by the viscosities of flowable resins. Hence, we recommend that, as per the site and morphology in clinical practice, the proper viscosity of flowable resin should be used for different cavities. Although this is the only study to confirm the three-body wear of flowable resins using a toothbrush abrasion test with toothpaste, previous studies reported that the same resin composite demonstrated a different behavior between two- and three-body wear [47]. In the future, a localized wear test must be conducted to examine the relationship between the viscosity and wear resistance of flowable resin composites, thus reflecting the clinical condition better.

## 5. Conclusions

Based on the limitations of this study, we can conclude the following:

1. We reported no significant differences among the flowable resins used in this study after 10,000, 20,000, 30,000, or 40,000 strokes.
2. We identified significant differences in viscosity among all flowable resin composites except between P03 and X03 at a shear rate of 1.0 s^−1^ and between F02 and P00 and between P03 and X03 at a shear rate of 2.0 s^−1^.
3. We detected no significant correlation between the viscosity and amount of wear of the flowable resin composites after the toothbrush abrasion test.

## Figures and Tables

**Table 1:** Flowable resin composites used in this study.

Materials	Abbreviation	Lot No.	Composition	Filler Contents (wt%)	Mean Filler Size (µm)	Manufacturer
BEAUTIFIL Flow F02	F02	061918	Bis-GMA, TEGDMA, long chain crosslinking monomer, photoinitiator	54.5	0.8	
BEAUTIFIL Flow F10	F10	061905	Bis-GMA, TEGDMA, long chain crosslinking monomer, photoinitiator	53.8	0.8	
BEAUTIFIL Flow Plus F00	P00	041919	Bis-GMA, TEGDMA, photoinitiator	67.3	0.8	Shofu Inc.
BEAUTIFIL Flow Plus F03	P03	061918	Bis-GMA, TEGDMA, photoinitiator	66.8	0.8	
BEAUTIFIL Flow Plus X F00	X00	071904	Bis-GMA, Bis-MPEPP, TEGDMA, Photoinitiator	63.7	0.4	
BEAUTIFIL Flow Plus X F03	X03	061904	Bis-GMA, Bis-MPEPP, TEGDMA, Photoinitiator	63.4	0.4	

Bis-GMA: bisphenol A-glycidyl methacrylate, TEGDMA: Triethylene glycol dimethacrylate; Bis-MPEPP: 2, 2’-bis (4-methacryloxy polyethoxyphenyl) propane.
